# Factors affecting the use of herbal medicines for weight loss in overweight and obese adolescents

**DOI:** 10.3389/fped.2023.1166786

**Published:** 2023-04-26

**Authors:** Mi Hong Yim, Boram Lee

**Affiliations:** ^1^ Digital Health Research Division, Korea Institute of Oriental Medicine, Daejeon, Republic of Korea; ^2^ KM Science Research Division, Korea Institute of Oriental Medicine, Daejeon, Republic of Korea

**Keywords:** herbal medicine, pediatric obesity, Korea youth risk behavior web-based survey, andersen's behavioral model, real-world data

## Abstract

**Objectives:**

Herbal medicine (HM) is widely used to treat obesity in adolescents worldwide since the currently available interventions have low compliance and lack long-term effects and safety data. This study aimed to analyze the factors affecting HM use for weight loss in overweight and obese adolescents.

**Methods:**

A total of 46,336 adolescents were included in this cross-sectional study based on the Korea Youth Risk Behavior Web-Based Survey. Three models of HM use for weigh loss were developed by sequentially adding predisposing, enabling, and need factors according to Andersen's model using multiple logistic regression analyses considering the complex sampling design.

**Results:**

Male and female high school students and students from low perceived household economic status were less likely to use HM for weight loss. Students whose fathers had a college degree or higher, depressed mood, and two or more chronic allergic diseases were more likely to use HM. Male students who perceived their body image as fat or very fat tended to use HM less than those who perceived their body image as very thin, thin, or moderate. Obese female students tended to use HM more than overweight female students.

**Conclusion:**

These results can be used as the bases to promote HM use, provide ideas for future research, and strengthen the health insurance coverage extension for weight loss interventions.

## 1. Introduction

Over the past few decades, the rapid increase in the number of adolescents that are overweight or obese has become a major public health problem (1). Recent data shows that the number of children and adolescents with obesity worldwide has increased dramatically over the past 40 years, with a ten-fold increase in girls and a twelve-fold increase in boys, highlighting the urgent need for effective programs and policies to address this growing epidemic (2). Additionally, about 70%–80% of adolescents with obesity had obesity in adulthood (3). Adolescent obesity can also lead to an increased risk of chronic diseases, such as metabolic syndrome, increasing the worldwide socio-economic burden (4).

Currently, lifestyle interventions, including exercise, dietary, and behavioral therapy, are recommended for adolescents with obesity (5, 6). However, it has been reported that patients did not achieve weight loss in a long-term real-world clinical study (7) and that severely obese adolescents were reluctant to practice the lifestyle interventions (8). Liraglutide and bariatric surgery is also considered for severely obese adolescents; however, long-term safety and efficacy data are lacking (1, 9, 10). Due to these limited treatment options, there is a demand for complementary and integrative medicine (CIM) (11).

Herbal medicine (HM) is one of the most common CIM therapies for weight loss worldwide (11, 12). It has been reported that people prefer HM as a treatment option due to reasons such as dissatisfaction with conventional treatment, past positive experiences, and family traditions (13). HM can reduce weight by suppressing the appetite, promoting lipid metabolism, inhibiting pancreatic lipase activity, increasing lipolysis, and preventing adipogenesis (14). In addition, several studies on HM use and the comparative effectiveness and safety of HM in obese adolescent patients, have been conducted (12, 15). However, studies on the factors affecting HM use for weight loss in adolescents have not been conducted yet.

Recently, the demand for high-quality healthcare services has gradually increased, and the government's healthcare policies to provide them have also expanded. Policies established based on scientific evidence using real-world data (RWD) analyses at the national level have gradually increased worldwide (16). Analyzing the factors influencing the use of HM for weight loss using RWD can be used as a basis for establishing policies for efficient resource allocation, clinicians, and researchers by identifying the HM usage and presenting data that can increase the usage rate.

Therefore, this study aimed to analyze the factors affecting HM use for weight loss in overweight and obese adolescents using the Korea Youth Risk Behavior Web-Based Survey (KYRBS) and to use the data for clinical use, future research plans, and policy establishment.

## 2. Methods

### 2.1. Data source and study participants

This study used the KYRBS, a national school-based representative cross-sectional survey conducted annually by the Korea Disease Control and Prevention Agency, to evaluate the health risk behaviors of Korean adolescents since 2005 (17). The KYRBS was designed to represent Korean school adolescents using a complex sampling design, including multistage sampling, stratification, and clustering (17). The KYRBS was collected using anonymous self-administered web-based questionnaires with administrative support from the Ministry of Education and had a response rate of over 95% (17). The questionnaires included questions on health risk behaviors such as socioeconomic status, dietary behaviors, physical activity, obesity and weight control, tobacco and alcohol use, mental health, and allergic disease diagnoses. School students aged 12 to 18 completed the questionnaires online in a school computer laboratory during one class period following the instructions of a trained teacher on the survey procedure after checking online informed consent (17). The reports, microdata, and publications of the KYRBS are available to the public on the KYRBS website [http://www.kdca.go.kr/yhs/]. Since the KYRBS did not collect any identifiable information about human participants and was publicly accessible, it was not considered human subject research. The Korea Institute of Oriental Medicine review board approved this study as an exemption from review (I-2206/006-003).

**Figure 1:**
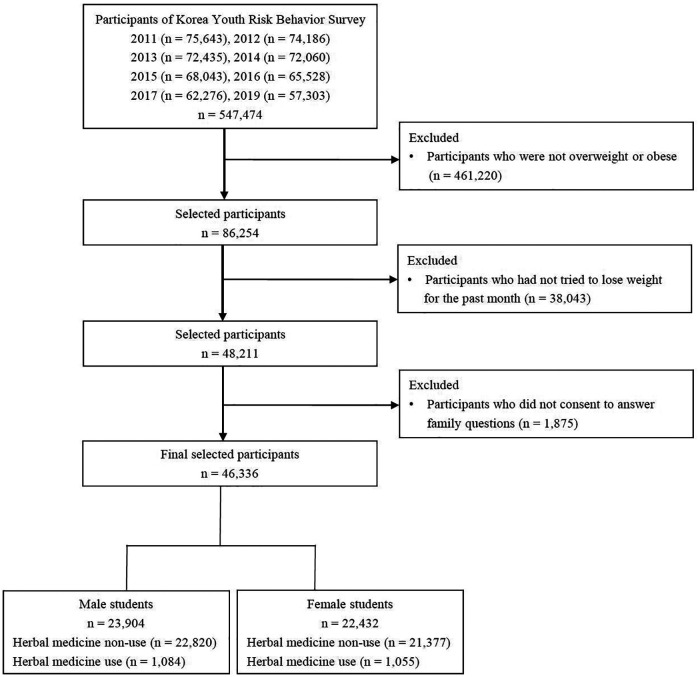
Flowchart for study participant selection.

The study included overweight and obese adolescents who had tried any method to lose weight over the past 30 days. We used KYRBS from 2011 to 2019, excluding 2018, because questions on how to lose weight were not included in the 2018 KYRBS and after 2020. A total of 547,474 adolescents participated in the 2011 to 2017 and 2019 KYRBS. We excluded 461,220 adolescents who were not overweight or obese and 38,043 adolescents who had not tried to lose weight over the past 30 days. Additionally, 1,875 adolescents who did not consent to answer family questions were excluded. In total, 46,336 adolescents were included (Figure 1).

### 2.2. Outcome and explanatory variables

The study's outcome was whether overweight and obese adolescents used HM for weight loss. HM use for weight loss was based on two questions: “Have you made efforts to control your weight over the past 30 days” and “What methods have you used to lose weight.” The overweight and obese adolescents who answered “I have tried to lose weight” to the first question and “I have used HM” to the second question were classified as the HM use group. The adolescents who answered other weight control methods (regular exercise, fasting for at least 24 h, eating less, prescription weight loss medication, over-the-counter weight loss medication, laxatives or diuretics, vomiting after eating, monotrophic diet, or dietary supplements) to the second question were classified as the HM non-use group. Overweight adolescents were defined as adolescents with a body mass index (BMI) at between the 85th and the 95th percentile, and obese adolescents were defined as adolescents with a BMI at or above the 95th percentile for age and sex.

The explanatory variables were selected based on Andersen's theoretical framework to investigate the factors affecting the use of HM for weight loss in overweight and obese adolescents (18, 19). Andersen's Behavioral Model of Health Services Use has been frequently applied to substantial studies to determine the effects of health services use (19). The explanatory variables included demographic and socio-economic characteristics, mental health, quality of life, and health behaviors. According to Andersen's model, we classified the explanatory variables into three categories of factors: predisposing, enabling, and need. Predisposing factors represented individual characteristics that existed prior to health services use (20), including school grade, region, and perceived academic record. Enabling factors indicated the resources for making health services use possible (21, 22), including residential areas, current residence type, perceived household economic status, and father's and mother's education level. Need factors refer to the perceived physical, mental, and diagnosed health statuses (23), including variables regarding health status (perceived health status, perceived body image, BMI, and sleep satisfaction), dietary behaviors (breakfast and fast food consumption for the past seven days, and nutrition education for the past 12 months), mental health (perceived stress, suicidal ideation, and depressed mood), health behaviors (alcohol and tobacco use for the past 30 days, and physical activity), and disease (number of chronic allergic diseases, including asthma, allergic rhinitis, and atopic dermatitis).

### 2.3. Statistical analyses

Since the KYRBS used a complex, multistage stratified, cluster sampling design to represent the target population, certain groups of participants may be oversampled. Therefore, the sample weights were generated in the KYRBS to correct for unequal chances in participant selection due to the complex sampling design and to adjust for non-response and post-stratification. In all the statistical analyses, the sample weights were integrated and used so that the results were representative of Korean school adolescents. To take account of sex-specific differences in several explanatory variables, including mental health and health behaviors, all statistical analyses were performed according to sex.

To compare general characteristics between the HM use and HM non-use groups, we used general linear model analyses for continuous variables and chi-squared tests with Rao-Scott correction for the categorical variables. The results are summarized as the mean ± standard error for the continuous variables and as the frequency (weighted column percentage) for the categorical variables. To investigate the association between HM use for weight loss and the individual variables of predisposing, enabling, and need factors, simple logistic regression analyses were performed for each sex. The results are indicated as crude odds ratios and 95% confidence intervals (CIs). To evaluate the association between HM use for weight loss and the combined variables of predisposing, enabling, and need factors, three multiple logistic regression models were constructed by sequentially adding the three factors conceptually classified in Andersen's model for each sex. Model 1 comprised the combined variables of predisposing factors. Model 2 comprised the combined variables of predisposing and enabling factors. Model 3 comprised the combined variables of predisposing, enabling, and need factors. The results are reported as adjusted odds ratios (aORs) and 95% CIs. All statistical analyses were performed using complex samples procedure in IBM SPSS Statistics for Windows, version 28.0.0.0 (IBM Corp., Armonk, NY, USA). All statistical tests were applied with a significance level of 0.05 and a two-tailed test.

## 3. Results

A total of 46,336 overweight and obese adolescents (23,904 male and 22,432 female students) were included. Of them, 2,139 adolescents (1,084 male and 1,055 female students) reported using HM for weight loss. In both the male and female students, there were significant differences between the HM use and HM non-use groups in school grade, perceived household economic status, education level of their father and mother, perceived health status and stress, suicidal ideation, depressed mood, tobacco use, diagnoses of asthma, allergic rhinitis, and atopic dermatitis, and the number of chronic allergic diseases diagnosed. In the male students, significant differences between groups were observed in height, weight, perceived body image, fast food consumption, and nutrition education. In the female students, significant differences between groups were found in residential areas, BMI, sleep satisfaction, and alcohol use (Table 1).

**Table 1:** General characteristics of participants between herbal medicine use and non-use groups.

Variables	Male students				Female students			
	Herbal medicine				Herbal medicine			
	Total	Non-use	Use	*P* value	Total	Non-use	Use	*P* value
Number of participants	23,904	22,820	1,084	NA	22,432	21,377	1,055	NA
**Predisposing factors**								
School grade				<.001				.007
Middle school	12,149 (47.4)	11,476 (46.8)	673 (59.5)		11,379 (48.1)	10,810 (47.9)	569 (52.5)	
High school	11,755 (52.6)	11,344 (53.2)	411 (40.5)		11,053 (51.9)	10,567 (52.1)	486 (47.5)	
Region				.090				.094
Seoul/Gyeonggi/Incheon	9,339 (45.6)	8,917 (45.6)	422 (45.6)		8,815 (44.8)	8,363 (44.7)	452 (48.1)	
Gangwon	945 (3.3)	901 (3.3)	44 (3.9)		908 (3.6)	869 (3.6)	39 (3.5)	
Daejeon/Chungcheong/Sejong	3,195 (11.2)	3,071 (11.3)	124 (8.9)		3,014 (11.4)	2,882 (11.4)	132 (10.9)	
Gwangju/Jeolla/Jeju	3,603 (12.6)	3,449 (12.6)	154 (12)		3,607 (13.7)	3,466 (13.8)	141 (11.2)	
Busan/Daegu/Ulsan/Gyeongsang	6,822 (27.3)	6,482 (27.2)	340 (29.7)		6,088 (26.5)	5,797 (26.5)	291 (26.3)	
Perceived academic record				.366				.417
High	8,114 (33.5)	7,739 (33.5)	375 (34.5)		6,594 (29)	6,301 (29.1)	293 (27.2)	
Middle	6,668 (28)	6,351 (28)	317 (29.3)		6,197 (27.8)	5,895 (27.7)	302 (29.2)	
Low	9,122 (38.5)	8,730 (38.6)	392 (36.2)		9,641 (43.2)	9,181 (43.2)	460 (43.6)	
**Enabling factors**								
Residential areas				.287				.008
Rural areas	2,459 (6.9)	2,364 (7)	95 (5.8)		2,484 (7.5)	2,379 (7.6)	105 (6.2)	
Metropolitan cities	10,602 (43.7)	10,110 (43.7)	492 (43.6)		9,507 (42.2)	9,009 (42)	498 (46.7)	
Small/medium cities	10,843 (49.4)	10,346 (49.4)	497 (50.7)		10,441 (50.3)	9,989 (50.5)	452 (47.1)	
Residence type				.650				.558
Living with family	22,883 (96.1)	21,841 (96.1)	1,042 (96.4)		21,491 (96.5)	20,489 (96.5)	1,002 (96.1)	
Living with relatives/dormitory/etc.	1,021 (3.9)	979 (3.9)	42 (3.6)		941 (3.5)	888 (3.5)	53 (3.9)	
Perceived household economic status				<.001				<.001
High	9,344 (39.2)	8,806 (38.8)	538 (49.1)		6,243 (28.1)	5,830 (27.5)	413 (40.2)	
Middle	10,239 (42.6)	9,839 (42.8)	400 (37.8)		10,818 (47.9)	10,420 (48.4)	398 (37.3)	
Low	4,321 (18.2)	4,175 (18.4)	146 (13)		5,371 (24)	5,127 (24)	244 (22.6)	
Father's education level				<.001				<.001
≤high school graduate	8,232 (33.9)	7,938 (34.2)	294 (26.2)		9,064 (39.4)	8,708 (39.8)	356 (32.2)	
≥college graduate	10,544 (45.8)	9,972 (45.4)	572 (55.3)		8,495 (40.1)	8,011 (39.6)	484 (48.9)	
Unknown/fatherless	5,128 (20.3)	4,910 (20.4)	218 (18.5)		4,873 (20.5)	4,658 (20.6)	215 (18.8)	
Mother's education level				<.001				<.001
≤high school graduate	9,514 (40.1)	9,152 (40.5)	362 (33.6)		10,906 (48.5)	10,464 (48.9)	442 (41.6)	
≥college graduate	9,199 (39.6)	8,691 (39.1)	508 (48.5)		7,347 (34.2)	6,897 (33.7)	450 (44.5)	
Unknown/motherless	5,191 (20.3)	4,977 (20.4)	214 (17.9)		4,179 (17.2)	4,016 (17.4)	163 (14)	
**Need Factors**								
Height	170.19 ± 0.14	171.24 ± 0.07	169.14 ± 0.27	<.001	160.33 ± 0.1	160.47 ± 0.04	160.19 ± 0.19	.162
Weight	78.17 ± 0.18	79.23 ± 0.09	77.1 ± 0.34	<.001	65.5 ± 0.11	65.36 ± 0.05	65.65 ± 0.22	.205
Perceived health status				.011				<.001
Very good/good	16,861 (70.5)	16,132 (70.7)	729 (66.1)		13,795 (61.3)	13,188 (61.5)	607 (57.7)	
Fair	5,520 (23)	5,247 (22.8)	273 (26.1)		6,666 (29.8)	6,346 (29.8)	320 (29.8)	
Poor/very poor	1,523 (6.5)	1,441 (6.4)	82 (7.9)		1,971 (9)	1,843 (8.8)	128 (12.6)	
Perceived body image				.007				.961
Very thin/thin/moderate	1,092 (4.5)	1,018 (4.4)	74 (6.3)		727 (3.1)	692 (3.1)	35 (3.2)	
Fat/very fat	22,812 (95.5)	21,802 (95.6)	1,010 (93.7)		21,705 (96.9)	20,685 (96.9)	1,020 (96.8)	
BMI				.452				<.001
Overweight	10,978 (46.1)	10,505 (46.2)	473 (44.9)		12,715 (57)	12,210 (57.5)	505 (48.1)	
Obese	12,926 (53.9)	12,315 (53.8)	611 (55.1)		9,717 (43)	9,167 (42.5)	550 (51.9)	
Sleep satisfaction				.338				.022
Very enough/enough	8,311 (34.1)	7,953 (34.1)	358 (32.3)		4,793 (21.1)	4,569 (21.1)	224 (21.2)	
Moderate	7,950 (33.6)	7,600 (33.7)	350 (33.4)		7,226 (32)	6,914 (32.2)	312 (28.1)	
Not enough/not enough at all	7,643 (32.3)	7,267 (32.2)	376 (34.4)		10,413 (46.9)	9,894 (46.8)	519 (50.8)	
Breakfast consumption				.553				.324
≤2 times/week	6,742 (28.1)	6,450 (28.2)	292 (26.8)		6,850 (30.4)	6,505 (30.2)	345 (32.5)	
3–6 times/week	7,541 (31.5)	7,203 (31.6)	338 (31.2)		8,425 (37.7)	8,038 (37.8)	387 (36.1)	
Everyday	9,621 (40.4)	9,167 (40.3)	454 (42)		7,157 (31.9)	6,834 (31.9)	323 (31.3)	
Fast food consumption				.015				.124
None/week	7,464 (30.9)	7,130 (30.9)	334 (30.7)		7,353 (32.3)	7,014 (32.3)	339 (32.5)	
1–2 times/week	13,271 (55.9)	12,702 (56.1)	569 (53)		12,603 (56.6)	12,026 (56.7)	577 (54.4)	
≥3 times/week	3,169 (13.2)	2,988 (13.1)	181 (16.3)		2,476 (11.1)	2,337 (11)	139 (13)	
Soft drink consumption				.152				.620
None/week	5,008 (21)	4,794 (21.1)	214 (19.3)		7,193 (32.2)	6,876 (32.2)	317 (31.2)	
1–2 times/week	11,566 (48.5)	11,064 (48.6)	502 (47.6)		11,112 (49.6)	10,579 (49.6)	533 (49.4)	
≥3 times/week	7,330 (30.4)	6,962 (30.3)	368 (33.1)		4,127 (18.3)	3,922 (18.2)	205 (19.4)	
Vegetable consumption				.432				.957
≤2 times/week	3,379 (14.1)	3,227 (14.1)	152 (13.7)		4,051 (18.2)	3,867 (18.2)	184 (18)	
3–6 times/week	8,915 (37.3)	8,499 (37.2)	416 (39.3)		8,583 (38.4)	8,173 (38.3)	410 (38.8)	
Everyday	11,610 (48.6)	11,094 (48.7)	516 (47)		9,798 (43.4)	9,337 (43.4)	461 (43.2)	
Nutrition education				.001				.075
No	12,493 (53.5)	11,964 (53.8)	529 (48.4)		13,289 (60.3)	12,688 (60.4)	601 (57.4)	
Yes	11,411 (46.5)	10,856 (46.2)	555 (51.6)		9,143 (39.7)	8,689 (39.6)	454 (42.6)	
Perceived stress				<.001				<.001
Low/none	5,156 (21.2)	4,961 (21.4)	195 (17.2)		2,514 (11)	2,418 (11.1)	96 (9.8)	
Moderate	10,281 (43.1)	9,856 (43.3)	425 (39.8)		8,382 (37.7)	8,054 (38.1)	328 (30.3)	
Very high/high	8,467 (35.7)	8,003 (35.3)	464 (43)		11,536 (51.3)	10,905 (50.8)	631 (59.9)	
Suicidal ideation				<.001				<.001
No	20,927 (87.3)	20,049 (87.6)	878 (81.3)		17,688 (78.7)	16,919 (79)	769 (72.9)	
Yes	2,977 (12.7)	2,771 (12.4)	206 (18.7)		4,744 (21.3)	4,458 (21)	286 (27.1)	
Depressed mood				<.001				<.001
No	18,288 (76.1)	17,537 (76.5)	751 (68)		14,523 (64.8)	13,939 (65.2)	584 (55.5)	
Yes	5,616 (23.9)	5,283 (23.5)	333 (32)		7,909 (35.2)	7,438 (34.8)	471 (44.5)	
Alcohol use				.085				.033
No	19,101 (79.2)	18,212 (79.1)	889 (81.5)		18,971 (84.4)	18,114 (84.6)	857 (82)	
Yes	4,803 (20.8)	4,608 (20.9)	195 (18.5)		3,461 (15.6)	3,263 (15.4)	198 (18)	
Tobacco use				.013				.007
No	17,246 (71.3)	16,427 (71.2)	819 (75)		19,455 (86.8)	18,583 (86.9)	872 (83.9)	
Yes	6,658 (28.7)	6,393 (28.8)	265 (25)		2,977 (13.2)	2,794 (13.1)	183 (16.1)	
Physical activity				.053				.102
None/week	2,410 (10.4)	2,318 (10.5)	92 (8.9)		5,950 (27)	5,702 (27.2)	248 (23.5)	
1–2 times/week	8,791 (36.8)	8,357 (36.6)	434 (40.9)		9,703 (43.3)	9,240 (43.2)	463 (45.5)	
3–4 times/week	7,317 (30.6)	7,009 (30.7)	308 (29)		4,598 (20.1)	4,369 (20.1)	229 (20.9)	
≥5 times/week	5,386 (22.2)	5,136 (22.2)	250 (21.2)		2,181 (9.6)	2,066 (9.5)	115 (10.1)	
Asthma				<.001				<.001
No	23,153 (96.9)	22,122 (97)	1,031 (95)		21,929 (97.8)	20,921 (97.9)	1,008 (95.8)	
Yes	751 (3.1)	698 (3)	53 (5)		503 (2.2)	456 (2.1)	47 (4.2)	
Allergic rhinitis				<.001				<.001
No	19,631 (81.7)	18,784 (81.9)	847 (76.8)		18,231 (80.7)	17,429 (80.9)	802 (76)	
Yes	4,273 (18.3)	4,036 (18.1)	237 (23.2)		4,201 (19.3)	3,948 (19.1)	253 (24)	
Atopic dermatitis				.006				.001
No	22,241 (92.9)	21,253 (93)	988 (90.6)		20,305 (90.5)	19,383 (90.7)	922 (87.5)	
Yes	1,663 (7.1)	1,567 (7)	96 (9.4)		2,127 (9.5)	1,994 (9.3)	133 (12.5)	
Number of chronic allergic disease				<.001				<.001
0	18,223 (75.8)	17,441 (76.1)	782 (70.5)		16,651 (73.7)	15,949 (74.1)	702 (66.8)	
1	4,775 (20.3)	4,546 (20.2)	229 (22.4)		4,824 (22)	4,541 (21.8)	283 (26.7)	
2 or 3	906 (3.9)	833 (3.7)	73 (7)		957 (4.3)	887 (4.1)	70 (6.5)	

For continuous variables, values are presented as mean ± standard error and *P* values were obtained using general linear model analyses. For categorical variables, values are presented as unweighted frequency (weighted column proportion) and *P* values were obtained using chi-squared tests with Rao-Scott correction. Sample weights were integrated and used, in all analyses performed for male and female students, respectively.

BMI, body mass index; NA, not applicable.

**Figure 2:**
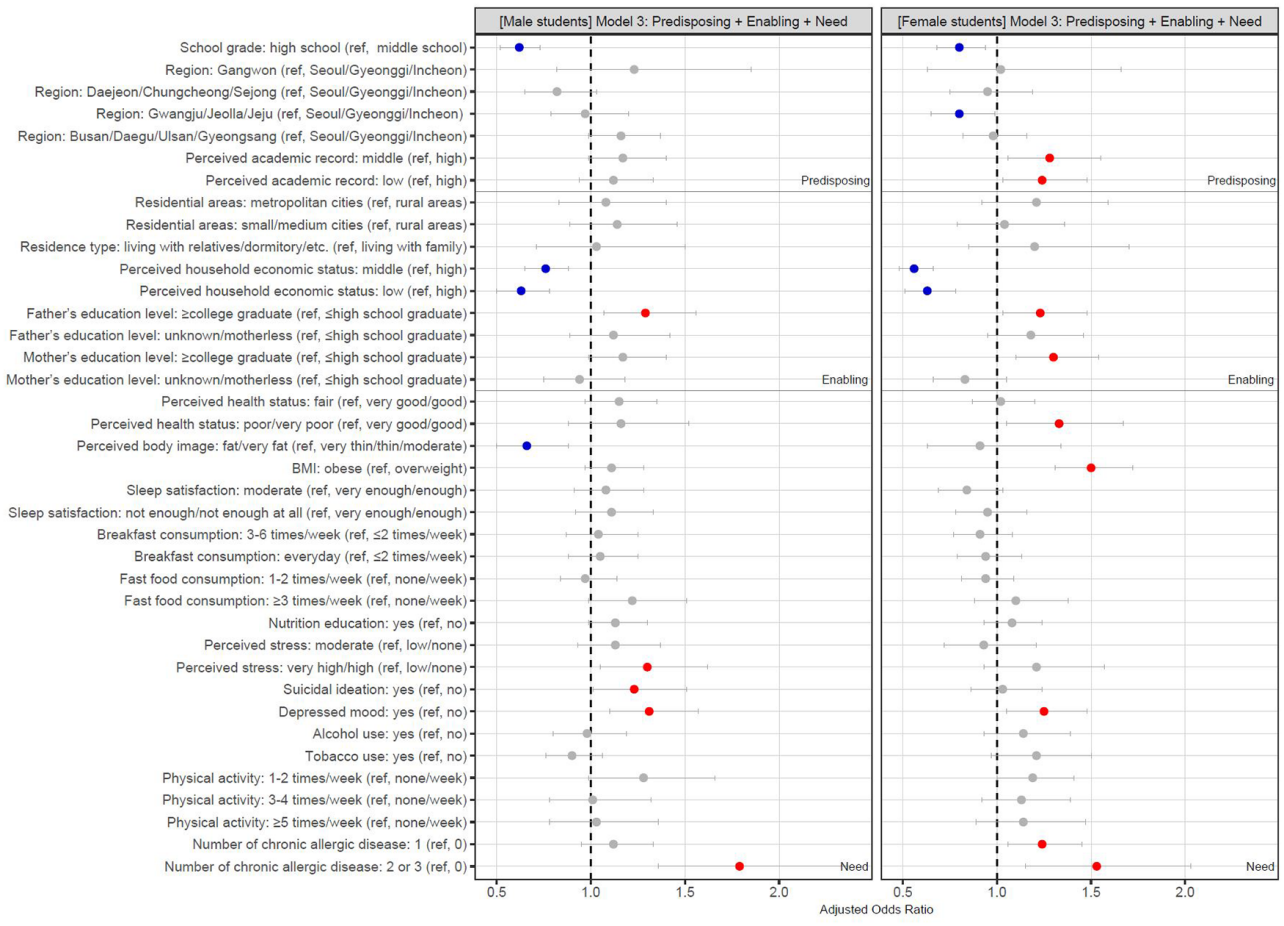
Adjusted odds ratios and 95% confidence interval in fully adjusted model 3. HM, herbal medicine; ref, reference group. The adjusted odds ratios and 95% confidence intervals of fully adjusted model 3 are visually presented to show the association between HM use for weight loss and the combined variables of predisposing, enabling, and need factors. Red dot indicates that the group is more likely to use HM than the reference group. Blue dot indicates that the group is less likely to use HM than the reference group. Grey dot indicates that the group is not significantly related to the reference group.

For the male students, crude analyses showed that HM use for weight loss was significantly associated with school grade, region, perceived household economic status, father's and mother's education level, perceived health status, perceived body image, fast food consumption, nutrition education, perceived stress, suicidal ideation, depression, tobacco use, physical activity, and the number of chronic allergic diseases. However, after sequentially adjusting for predisposing, enabling, and need factors, only some of these variables were related to HM use. In model 1, which included the predisposing factors, there were significant associations of HM use with school grade and region. In model 2, which included predisposing and enabling factors, significant associations were found between HM use and school grade, perceived household economic status, and father's education level. In model 3, which included predisposing, enabling, and need factors, HM use was significantly associated with school grade, perceived household economic status, father's education level, perceived body image, perceived stress, suicidal ideation, depression, and the number of chronic allergic diseases. In the fully adjusted model 3, high school students were less likely to use HM compared to middle school students (aOR [95% CI], 0.62 [0.52–0.73]). Students who perceived their household economic status as middle/low were less likely to use HM compared with those who perceived their household economic status as high (middle, 0.76 [0.65–0.88]; low, 0.63 [0.5–0.78]). Students whose fathers had a college-level or higher degree were more likely to use HM than those whose fathers had a high school-level or lower degree [1.29 (1.07–1.56)]. Students who perceived their body as being fat/very fat tended to use HM less than those who perceived their body as being very thin/thin/moderate [0.66 (0.5–0.88)]. Students under very high/high stress levels were more likely to use HM than those under low/no stress levels [1.3 (1.05–1.62)]. Students with suicidal ideation [1.23 (1.01–1.51)] and depressed mood [1.31 (1.1–1.57)] were more likely to use HM compared to those without. Students with two or three chronic allergic diseases tended to use HM more than those without [1.79 (1.36–2.36)] (Figure 2, Table 2).

**Table 2:** Association of herbal medicine use with predisposing, enabling, and need factors in male students.

Variables	Crude Model		Model 1		Model 2		Model 3	
	cOR (95% CI)	*P* value	aOR (95% CI)	*P* value	aOR (95% CI)	*P* value	aOR (95% CI)	*P* value
**Predisposing factors**								
**School grade**								
Middle school	1 [Reference]		1 [Reference]		1 [Reference]		1 [Reference]	
High school	0.6 (0.52–0.69)	<.001	0.6 (0.52–0.69)	<.001	0.62 (0.53–0.71)	<.001	0.62 (0.52–0.73)	<.001
**Region**								
Seoul/Gyeonggi/Incheon	1 [Reference]		1 [Reference]		1 [Reference]		1 [Reference]	
Gangwon	1.19 (0.82–1.73)	.371	1.18 (0.81–1.72)	.395	1.22 (0.82–1.8)	.327	1.23 (0.82–1.85)	.311
Daejeon/Chungcheong/Sejong	0.79 (0.63–0.99)	.042	0.79 (0.63–0.99)	.040	0.82 (0.65–1.04)	.097	0.82 (0.65–1.03)	.089
Gwangju/Jeolla/Jeju	0.95 (0.78–1.17)	.648	0.95 (0.78–1.17)	.649	0.96 (0.78–1.18)	.689	0.97 (0.79–1.2)	.799
Busan/Daegu/Ulsan/Gyeongsang	1.09 (0.94–1.28)	.264	1.09 (0.93–1.27)	.289	1.13 (0.96–1.32)	.131	1.16 (0.99–1.37)	.061
**Perceived academic record**								
High	1 [Reference]		1 [Reference]		1 [Reference]		1 [Reference]	
Middle	1.02 (0.86–1.2)	.858	1.06 (0.89–1.26)	.524	1.18 (0.99–1.4)	.065	1.17 (0.99–1.4)	.070
Low	0.91 (0.78–1.07)	.258	0.95 (0.81–1.12)	.563	1.15 (0.97–1.36)	.102	1.12 (0.94–1.33)	.210
**Enabling factors**								
**Residential areas**								
Rural areas	1 [Reference]		NA	NA	1 [Reference]		1 [Reference]	
Metropolitan cities	1.2 (0.94–1.53)	.140	NA	NA	1.1 (0.84–1.42)	.493	1.08 (0.83–1.4)	.577
Small/medium cities	1.23 (0.97–1.57)	.089	NA	NA	1.16 (0.91–1.49)	.237	1.14 (0.89–1.46)	.306
**Residence type**								
Living with family	1 [Reference]		NA	NA	1 [Reference]		1 [Reference]	
Living with relatives/dormitory/etc.	0.92 (0.64–1.32)	.652	NA	NA	1.13 (0.78–1.64)	.523	1.03 (0.71–1.5)	.869
**Perceived household economic status**								
High	1 [Reference]		NA	NA	1 [Reference]		1 [Reference]	
Middle	0.7 (0.6–0.81)	<.001	NA	NA	0.76 (0.66–0.89)	<.001	0.76 (0.65–0.88)	<.001
Low	0.56 (0.46–0.69)	<.001	NA	NA	0.67 (0.54–0.84)	<.001	0.63 (0.5–0.78)	<.001
**Father's education level**								
≤high school graduate	1 [Reference]		NA	NA	1 [Reference]		1 [Reference]	
≥college graduate	1.59 (1.35–1.87)	<.001	NA	NA	1.33 (1.1–1.6)	.003	1.29 (1.07–1.56)	.007
Unknown/fatherless	1.19 (0.97–1.45)	.092	NA	NA	1.13 (0.9–1.43)	.295	1.12 (0.89–1.42)	.315
**Mother's education level**								
≤high school graduate	1 [Reference]		NA	NA	1 [Reference]		1 [Reference]	
≥college graduate	1.49 (1.28–1.74)	<.001	NA	NA	1.18 (0.99–1.41)	.061	1.17 (0.99–1.4)	.073
Unknown/motherless	1.05 (0.87–1.28)	.595	NA	NA	0.91 (0.73–1.15)	.434	0.94 (0.75–1.18)	.583
**Need Factors**								
**Perceived health status**								
Very good/good	1 [Reference]		NA	NA	NA	NA	1 [Reference]	
Fair	1.22 (1.04–1.43)	.012	NA	NA	NA	NA	1.15 (0.97–1.35)	.103
Poor/very poor	1.3 (1.01–1.69)	.044	NA	NA	NA	NA	1.16 (0.88–1.52)	.288
**Perceived body image**								
Very thin/thin/moderate	1 [Reference]		NA	NA	NA	NA	1 [Reference]	
Fat/very fat	0.68 (0.52–0.9)	.007	NA	NA	NA	NA	0.66 (0.5–0.88)	.005
**BMI**								
Overweight	1 [Reference]		NA	NA	NA	NA	1 [Reference]	
Obese	1.05 (0.92–1.21)	.455	NA	NA	NA	NA	1.11 (0.97–1.28)	.132
**Sleep satisfaction**								
Very enough/enough	1 [Reference]		NA	NA	NA	NA	1 [Reference]	
Moderate	1.05 (0.89–1.24)	.573	NA	NA	NA	NA	1.08 (0.91–1.28)	.408
Not enough/not enough at all	1.13 (0.96–1.33)	.146	NA	NA	NA	NA	1.11 (0.92–1.33)	.265
**Breakfast consumption**								
≤2 times/week	1 [Reference]		NA	NA	NA	NA	1 [Reference]	
3–6 times/week	1.04 (0.87–1.24)	.693	NA	NA	NA	NA	1.04 (0.87–1.25)	.647
Everyday	1.1 (0.93–1.3)	.291	NA	NA	NA	NA	1.05 (0.88–1.25)	.568
**Fast food consumption**								
None/week	1 [Reference]		NA	NA	NA	NA	1 [Reference]	
1–2 times/week	0.95 (0.82–1.11)	.510	NA	NA	NA	NA	0.97 (0.84–1.14)	.748
≥3 times/week	1.25 (1.02–1.54)	.033	NA	NA	NA	NA	1.22 (0.99–1.51)	.058
**Nutrition education**								
No	1 [Reference]		NA	NA	NA	NA	1 [Reference]	
Yes	1.24 (1.09–1.42)	.001					1.13 (0.99–1.3)	.075
**Perceived stress**								
Low/none	1 [Reference]		NA	NA	NA	NA	1 [Reference]	
Moderate	1.14 (0.94–1.38)	.170	NA	NA	NA	NA	1.13 (0.93–1.37)	.234
Very high/high	1.52 (1.26–1.83)	<.001	NA	NA	NA	NA	1.3 (1.05–1.62)	.016
**Suicidal ideation**								
No	1 [Reference]		NA	NA	NA	NA	1 [Reference]	
Yes	1.63 (1.38–1.93)	<.001	NA	NA	NA	NA	1.23 (1.01–1.51)	.040
**Depressed mood**								
No	1 [Reference]		NA	NA	NA	NA	1 [Reference]	
Yes	1.53 (1.33–1.77)	<.001	NA	NA	NA	NA	1.31 (1.1–1.57)	.003
**Alcohol use**								
No	1 [Reference]		NA	NA	NA	NA	1 [Reference]	
Yes	0.86 (0.72–1.02)	.087	NA	NA	NA	NA	0.98 (0.8–1.19)	.807
**Tobacco use**								
No	1 [Reference]		NA	NA	NA	NA	1 [Reference]	
Yes	0.82 (0.7–0.96)	.014	NA	NA	NA	NA	0.9 (0.76–1.06)	.211
**Physical activity**								
None/week	1 [Reference]		NA	NA	NA	NA	1 [Reference]	
1–2 times/week	1.31 (1.02–1.68)	.032	NA	NA	NA	NA	1.28 (0.99–1.66)	.055
3–4 times/week	1.11 (0.86–1.44)	.438	NA	NA	NA	NA	1.01 (0.78–1.32)	.931
≥5 times/week	1.12 (0.86–1.47)	.405	NA	NA	NA	NA	1.03 (0.78–1.36)	.830
**Number of chronic allergic disease**								
0	1 [Reference]		NA	NA	NA	NA	1 [Reference]	
1	1.2 (1.01–1.41)	.035	NA	NA	NA	NA	1.12 (0.95–1.33)	.186
2 or 3	2.03 (1.56–2.65)	<.001	NA	NA	NA	NA	1.79 (1.36–2.36)	<.001

Crude models of HM use were obtained using simple logistic regression analyses for the individual variable of predisposing, enabling, and need factors in male students. Model 1 of HM use was obtained using multiple regression analyses for predisposing factors. Model 2 of HM use was obtained using multiple regression analyses for predisposing and enabling factors. Model 3 of HM use was obtained using multiple regression analyses for predisposing, enabling, and need factors. Sample weights were integrated and used, in all analyses for male students.

aOR, adjusted odds ratio; BMI, body mass index; CI, confidence interval; cOR, crude odds ratio; NA, not applicable.

For female students, crude analyses reported that HM use for weight loss was significantly associated with school grade, region, residential areas, perceived household economic status, father's and mother's education level, perceived health status, BMI, perceived stress, suicidal ideation, depressed mood, tobacco use, physical activity, and the number of chronic allergic diseases. After adjusting the same way as for the male students, only some of these variables were associated with HM use. In model 1, there were significant associations of HM use with school grade and region. In model 2, significant associations were observed between HM use and school grade, region, perceived academic record, perceived household economic status, and father's and mother's education level. In model 3, HM use was significantly associated with school grade, region, perceived academic record, perceived household economic status, father's and mother's education level, perceived health status, BMI, depression, and the number of chronic allergic diseases. In the fully adjusted model 3, high school students were less likely to use HM compared to middle school students [0.8 (0.68–0.94)]. Students living in Gwangju/Jeolla/Jeju tended to use HM less than those living in Seoul/Gyeonggi/Incheon [0.8 (0.65–0.99)]. Students with middle/low academic records were more likely to use HM compared with those with high records (middle, 1.28 [1.06–1.55]; low, 1.24 [1.03–1.48]). Students who perceived their household economic status as middle/low were less likely to use HM compared with those who perceived their household economic status as high (middle, 0.56 [0.48–0.66]; low, 0.63 [0.51–0.78]). Students whose parents had college-level or higher degrees were more likely to use HM than those whose parents had high school or lower degrees (father, 1.23 [1.03–1.48]; mother, 1.3 [1.1–1.54]). Students who perceived their health to be poor/very poor were more likely to use HM than those who perceived their health to be very good/good [1.33 (1.05–1.67)]. Obese students tended to use HM more than overweight students [1.5 (1.31–1.72)]. Students with depression were more likely to use HM than those without [1.25 (1.05–1.48)]. Students with one or two to three chronic allergic diseases were more likely to use HM compared to those without (one disease, 1.24 [1.06–1.45]; two to three diseases, 1.53 [1.15–2.03]) (Figure 2, Table 3).

**Table 3:** Association of herbal medicine use with predisposing, enabling, and need factors in female students.

Variables	Crude Model		Model 1		Model 2		Model 3	
	cOR (95% CI)	*P* value	aOR (95% CI)	*P* value	aOR (95% CI)	*P* value	aOR (95% CI)	*P* value
**Predisposing factors**								
**School grade**								
Middle school	1 [Reference]		1 [Reference]		1 [Reference]		1 [Reference]	
High school	0.83 (0.73–0.96)	.010	0.83 (0.72–0.95)	.008	0.84 (0.72–0.98)	.022	0.8 (0.68–0.94)	.007
**Region**								
Seoul/Gyeonggi/Incheon	1 [Reference]		1 [Reference]		1 [Reference]		1 [Reference]	
Gangwon	0.91 (0.58–1.43)	.670	0.9 (0.57–1.43)	.656	1.03 (0.63–1.67)	.916	1.02 (0.63–1.66)	.922
Daejeon/Chungcheong/Sejong	0.89 (0.71–1.11)	.299	0.89 (0.71–1.11)	.290	0.95 (0.76–1.2)	.662	0.95 (0.75–1.19)	.645
Gwangju/Jeolla/Jeju	0.75 (0.61–0.93)	.007	0.75 (0.61–0.93)	.007	0.8 (0.65–0.98)	.035	0.8 (0.65–0.99)	.040
Busan/Daegu/Ulsan/Gyeongsang	0.92 (0.78–1.09)	.346	0.92 (0.78–1.1)	.358	0.95 (0.8–1.13)	.553	0.98 (0.82–1.16)	.775
**Perceived academic record**								
High	1 [Reference]		1 [Reference]		1 [Reference]		1 [Reference]	
Middle	1.13 (0.94–1.35)	.198	1.14 (0.95–1.37)	.154	1.32 (1.09–1.58)	.004	1.28 (1.06–1.55)	.009
Low	1.08 (0.92–1.28)	.361	1.09 (0.93–1.29)	.296	1.36 (1.14–1.62)	<.001	1.24 (1.03–1.48)	.020
**Enabling factors**								
**Residential areas**								
Rural areas	1 [Reference]		NA	NA	1 [Reference]		1 [Reference]	
Metropolitan cities	1.37 (1.06–1.76)	.015	NA	NA	1.2 (0.91–1.58)	.194	1.21 (0.92–1.59)	.177
Small/medium cities	1.15 (0.89–1.48)	.298	NA	NA	1.04 (0.8–1.36)	.770	1.04 (0.79–1.36)	.780
**Residence type**								
Living with family	1 [Reference]		NA	NA	1 [Reference]		1 [Reference]	
Living with relatives/dormitory/etc.	1.1 (0.79–1.54)	.567	NA	NA	1.25 (0.89–1.77)	.204	1.2 (0.85–1.7)	.300
**Perceived household economic status**								
High	1 [Reference]		NA	NA	1 [Reference]		1 [Reference]	
Middle	0.53 (0.45–0.62)	<.001	NA	NA	0.55 (0.47–0.65)	<.001	0.56 (0.48–0.66)	<.001
Low	0.64 (0.54–0.77)	<.001	NA	NA	0.71 (0.58–0.87)	<.001	0.63 (0.51–0.78)	<.001
**Father's education level**								
≤high school graduate	1 [Reference]		NA	NA	1 [Reference]		1 [Reference]	
≥college graduate	1.52 (1.3–1.79)	<.001	NA	NA	1.21 (1.01–1.45)	.041	1.23 (1.03–1.48)	.023
Unknown/fatherless	1.13 (0.93–1.36)	.226	NA	NA	1.16 (0.93–1.44)	.180	1.18 (0.95–1.46)	.135
**Mother's education level**								
≤high school graduate	1 [Reference]		NA	NA	1 [Reference]		1 [Reference]	
≥college graduate	1.55 (1.33–1.8)	<.001	NA	NA	1.31 (1.11–1.55)	.002	1.3 (1.1–1.54)	.002
Unknown/motherless	0.94 (0.77–1.16)	.582	NA	NA	0.84 (0.66–1.06)	.136	0.83 (0.66–1.05)	.122
**Need Factors**								
**Perceived health status**								
Very good/good	1 [Reference]		NA	NA	NA	NA	1 [Reference]	
Fair	1.07 (0.91–1.24)	.423	NA	NA	NA	NA	1.02 (0.87–1.2)	.791
Poor/very poor	1.53 (1.23–1.9)	<.001	NA	NA	NA	NA	1.33 (1.05–1.67)	.016
**Perceived body image**								
Very thin/thin/moderate	1 [Reference]		NA	NA	NA	NA	1 [Reference]	
Fat/very fat	0.99 (0.69–1.43)	.961	NA	NA	NA	NA	0.91 (0.63–1.34)	.642
**BMI**								
Overweight	1 [Reference]		NA	NA	NA	NA	1 [Reference]	
Obese	1.46 (1.28–1.67)	<.001	NA	NA	NA	NA	1.5 (1.31–1.72)	<.001
**Sleep satisfaction**								
Very enough/enough	1 [Reference]		NA	NA	NA	NA	1 [Reference]	
Moderate	0.87 (0.72–1.06)	.156	NA	NA	NA	NA	0.84 (0.69–1.03)	.095
Not enough/not enough at all	1.08 (0.91–1.29)	.389	NA	NA	NA	NA	0.95 (0.78–1.16)	.590
**Breakfast consumption**								
≤2 times/week	1 [Reference]		NA	NA	NA	NA	1 [Reference]	
3–6 times/week	0.89 (0.75–1.05)	.156	NA	NA	NA	NA	0.91 (0.77–1.08)	.292
Everyday	0.91 (0.77–1.08)	.289	NA	NA	NA	NA	0.94 (0.79–1.13)	.525
**Fast food consumption**								
None/week	1 [Reference]		NA	NA	NA	NA	1 [Reference]	
1–2 times/week	0.95 (0.82–1.11)	.535	NA	NA	NA	NA	0.94 (0.81–1.09)	.394
≥3 times/week	1.18 (0.94–1.48)	.153	NA	NA	NA	NA	1.1 (0.88–1.38)	.410
**Nutrition education**								
No	1 [Reference]		NA	NA	NA	NA	1 [Reference]	
Yes	1.13 (0.99–1.3)	.082					1.08 (0.93–1.24)	.314
**Perceived stress**								
Low/none	1 [Reference]		NA	NA	NA	NA	1 [Reference]	
Moderate	0.9 (0.7–1.16)	.407	NA	NA	NA	NA	0.93 (0.72–1.21)	.584
Very high/high	1.33 (1.05–1.69)	.019	NA	NA	NA	NA	1.21 (0.93–1.57)	.157
**Suicidal ideation**								
No	1 [Reference]		NA	NA	NA	NA	1 [Reference]	
Yes	1.4 (1.2–1.63)	<.001	NA	NA	NA	NA	1.03 (0.86–1.24)	.749
**Depressed mood**								
No	1 [Reference]		NA	NA	NA	NA	1 [Reference]	
Yes	1.5 (1.31–1.72)	<.001	NA	NA	NA	NA	1.25 (1.05–1.48)	.010
**Alcohol use**								
No	1 [Reference]		NA	NA	NA	NA	1 [Reference]	
Yes	1.2 (1–1.44)	.053	NA	NA	NA	NA	1.14 (0.93–1.39)	.203
**Tobacco use**								
No	1 [Reference]		NA	NA	NA	NA	1 [Reference]	
Yes	1.28 (1.04–1.56)	.017	NA	NA	NA	NA	1.21 (0.97–1.5)	.086
**Physical activity**								
None/week	1 [Reference]		NA	NA	NA	NA	1 [Reference]	
1–2 times/week	1.22 (1.03–1.45)	.022	NA	NA	NA	NA	1.19 (1–1.41)	.051
3–4 times/week	1.2 (0.98–1.47)	.074	NA	NA	NA	NA	1.13 (0.92–1.39)	.251
≥5 times/week	1.23 (0.97–1.57)	.094	NA	NA	NA	NA	1.14 (0.89–1.47)	.299
**Number of chronic allergic disease**								
0	1 [Reference]		NA	NA	NA	NA	1 [Reference]	
1	1.36 (1.16–1.58)	<.001	NA	NA	NA	NA	1.24 (1.06–1.45)	.006
2 or 3	1.75 (1.33–2.3)	<.001	NA	NA	NA	NA	1.53 (1.15–2.03)	.003

Crude models of HM use were obtained using simple logistic regression analyses for the individual variable of predisposing, enabling, and need factors in male students. Model 1 of HM use was obtained using multiple regression analyses for predisposing factors. Model 2 of HM use was obtained using multiple regression analyses for predisposing and enabling factors. Model 3 of HM use was obtained using multiple regression analyses for predisposing, enabling, and need factors. Sample weights were integrated and used, in all analyses for female students.

aOR, adjusted odds ratio; BMI, body mass index; CI, confidence interval; cOR, crude odds ratio; NA, not applicable.

Common to male and female students, the variables associated with HM use for weight loss were school grade, perceived household economic status, father's education level, depressed mood, and number of chronic allergic diseases in the fully adjusted model 3. Perceived body image, perceived stress, and suicidal ideation were related to HM use only in male students and region, perceived academic record, mother's education level, perceived health status, and BMI were related to HM use only in female students.

## 4. Discussion

HM, a type of CIM, is actively used to treat obesity in adolescents worldwide since the currently available interventions have low compliance and lack long-term effects and safety data (1, 9, 10, 12). Studies on the usage, comparative effectiveness, and safety of HM have been conducted (12, 15). However, studies on the factors affecting HM use for weight loss in adolescents have not been conducted. This study investigated, for the first time, HM use for weight loss in overweight and obese adolescents and analyzed the predisposing, enabling, and need factors that affect HM use based on Andersen's behavior model using the KYRBS.

This study showed that after adjusting for all predisposing, enabling, and need factors, there was a significantly higher probability of HM use for weight loss in overweight and obese adolescents who were middle school students and who had higher perceived household economic status, father's education level, depressed mood, and two or more chronic allergic diseases in both male and female students. Looking at the differences by sex, only male students showed significantly less HM use when the perceived body image was fat or very fat compared with very thin, thin, or moderate. Male adolescents with very high perceived stress or suicidal ideation had significantly more HM use. Female students with middle or low perceived academic records, college graduate or higher mother's education level, poor or very poor perceived health status, and an obese BMI had significantly more HM use.

A previous survey in Germany found that the use of HM among children and adolescents declined with the increase in age (24). The determinants of HM use were young age, poor health status, and a family with a high social class (24). Another study identified the factors that affected Korean medicine health care use by privately insured people and found that patients with more chronic diseases used Korean medicine services more (25). A study analyzing the factors that affected Korean medicine healthcare use for treating functional dyspepsia found that participants with private health insurance and who were frequently or constantly stressed used the Korean medicine healthcare system more (26). These studies have similar results to our study. However, a prior study identified factors related to HM use in children and adolescents regardless of disease (24). A different study identified factors related to Korean medicine health care use, including HM, acupuncture, and moxibustion, in privately insured adults (25). The latter study also identified factors related to Korean medicine health care use in adult patients with functional dyspepsia (26). Our study was different because we limited the populations to overweight and obese adolescents that used HM for weight loss and analyzed the factors separately according to sex.

Based on modern medicine, HM can promote weight loss by reducing appetite, increasing lipid metabolism, inhibiting pancreatic lipase activity, promoting lipolysis, and preventing adipogenesis (14). While traditional medicine aims to balance the yin and yang by harmonizing the body, mind, and spirit (27), HM has multiple components and targets (28). Accordingly, various other systemic effects can be induced if a patient uses HM for weight loss. Several studies have reported that HM relieves stress and treats anxiety, depression, and suicide ideation (29–31). In addition, the effects of HM on chronic allergic diseases have been reported by previous studies (32–34). Furthermore, it has been reported that HM remarkably increases the neuroprotective effect and cognition and learning abilities through the induction and secretion of nerve growth factor (35). HM use has been reported to reduce the risk of comorbidities (36), and patients use HM with the expectation of treating comorbidities (37). Therefore, adolescents with high perceived stress, depressed mood, a high number of chronic allergic diseases, and low academic performance might use HM not only for weight loss but also for its therapeutic effect on comorbidities.

Interestingly, male students whose perceived body image was fat or very fat used HM significantly less compared with students whose perceived body image was very thin, thin, or moderate. To interpret this result, we checked the use of weight loss behaviors other than HM and the status of perceived body image in the HM use and HM non-use groups. As a result, overweight and obese adolescents whose perceived body image was very thin, thin, or moderate significantly used other weight loss methods, including regular exercise, fasting for at least 24 h, prescription weight loss medication, laxatives or diuretics, monotrophic diet, and vomiting after eating than those whose perceived body image was fat or very fat (Supplementary Meterial S1).

When analyzing HM use according to BMI, obese females used significantly more HM for weight loss than overweight females. To interpret this result, we checked the use of weight loss behaviors other than HM according to the status of BMI in both groups. As a result, obese adolescents tended to use weight loss methods more than overweight adolescents, except for regular exercise (in male students) and vomiting after eating (in female students); however, statistical significance could not be confirmed in some cases (Supplementary Meterial S2). Judging from these results, HM use differs according to the perceived body image and BMI classification, which seems not to be specific to HM use, but to other weight loss methods. Therefore, future studies should analyze the factors that affect weight loss efforts in overweight and obese adolescents. These studies will help provide data for effective weight loss practices in adolescents and related policy establishments.

While the KYRBS used for analysis did not address the effectiveness and safety of HM, previous studies have shown that HM can be more effective than placebo and lifestyle management in treating childhood obesity, with a low risk of serious adverse events (15). However, it is important to note that these studies have high risks of performance bias and unclear risks of selection and detection bias. Additionally, some patients taking HM have reported adverse events such as digestive and skin disorders, with a reported incidence of 6.6% (38). Therefore, it is crucial to conduct well-designed prospective clinical studies to further investigate the effectiveness and safety of HM for weight loss.

This study has the following limitations. First, KYRBS is a self-reported online survey conducted to identify the statuses and trends of adolescent health behaviors. In the case of HM use for weight loss, it is possible that adolescents responded by including over-the-counter herbal drugs and herbal health functional foods as well as HM prescriptions from Korean medicine clinics or hospitals. Differences according to these could not be confirmed due to the nature of the questionnaires since they were not developed for research purposes. Second, because KYRBS data are based on students' self-reports, responses may have been under or over-reported, especially height and weight. It has been reported that height is over-reported and weight is under-reported in the KYRBS (39). Therefore, it is possible that the definition of overweight and obesity based on the BMI percentiles in this study may not accurately represent overweight and obesity in adolescents. Finally, since this study is a cross-sectional analysis, the causal relationship of HM use for weight loss cannot be confirmed.

Nevertheless, this is the first study to identify the factors affecting HM use for weight loss in overweight and obese adolescents. In addition, the representativeness of the data was secured by using a large national representative database of Korean school adolescents. Furthermore, because of sex differences in obesity prevalence, treatment, and perception (40), we analyzed the data by sex. These results can be used to establish methods to promote HM use in clinical settings and can provide ideas for future research. Our study found that the use of HM for weight loss in adolescents was more prevalent among parents with higher education levels. This result is consistent with previous studies that show a positive association between education level and the use of CIM (41, 42), It has also been reported that providing more information on CIM promotes its use (43). Based on these findings, clinicians can promote the use of CIM, including HM, in clinical settings by providing sufficient information on the effectiveness and safety of HM for weight loss. This information can include evidence from previous studies, as well as potential risks and benefits. By doing so, clinicians can encourage informed decision-making and promote the use of HM in a safe and effective manner. In addition, given the high socioeconomic burden caused by obesity in adolescents, this study can be used as the bases for policymakers to strengthen the health insurance coverage extension for weight loss interventions.

## Data Availability

The original contributions presented in the study are included in the article/**Supplementary Material**, further inquiries can be directed to the corresponding author.
