# Supplementary material for: Factors affecting the use of herbal medicines for weight loss in overweight and obese adolescents
Source: Front Pediatr. 2023 Apr 26;11:1166786. doi: 10.3389/fped.2023.1166786 (PMC10171459; doi:10.3389/fped.2023.1166786)
Supplement: Supplementary file 1 [file Table1.docx]

**Supplement 1. Comparison of weight control methods according perceived body image**

| **Variables for weight control method** | **Male students** | | | | **Female students** | | | |
| --- | --- | --- | --- | --- | --- | --- | --- | --- |
|  | **Perceived body image** | | |  | **Perceived body image** | | |  |
|  | **Total** | **Very thin/ thin/ moderate** | **Fat/ very fat** | ***P* value** | **Total** | **Very thin/ thin/ moderate** | **Fat/ very fat** | ***P* value** |
| Regular exercise |  |  |  | <.001 |  |  |  | .001 |
| No | 4959 (20.9) | 167 (15.6) | 4792 (21.1) |  | 6222 (28) | 156 (22) | 6066 (28.2) |  |
| Yes | 18945 (79.1) | 925 (84.4) | 18020 (78.9) |  | 16210 (72) | 571 (78) | 15639 (71.8) |  |
| Fasting for at least 24 hours |  |  |  | .012 |  |  |  | .458 |
| No | 21914 (91.6) | 965 (89.4) | 20949 (91.7) |  | 19857 (88.8) | 624 (87.9) | 19233 (88.8) |  |
| Yes | 1990 (8.4) | 127 (10.6) | 1863 (8.3) |  | 2575 (11.2) | 103 (12.1) | 2472 (11.2) |  |
| Eating less |  |  |  | <.001 |  |  |  | <.001 |
| No | 4759 (19.6) | 306 (27.8) | 4453 (19.2) |  | 1775 (7.8) | 94 (13.8) | 1681 (7.6) |  |
| Yes | 19145 (80.4) | 786 (72.2) | 18359 (80.8) |  | 20657 (92.2) | 633 (86.2) | 20024 (92.4) |  |
| Prescription weight loss medication |  |  |  | .004 |  |  |  | .962 |
| No | 23451 (98) | 1053 (96.8) | 22398 (98.1) |  | 21751 (96.9) | 703 (96.9) | 21048 (96.9) |  |
| Yes | 453 (2) | 39 (3.2) | 414 (1.9) |  | 681 (3.1) | 24 (3.1) | 657 (3.1) |  |
| Over-the-counter weight loss medication |  |  |  | .001 |  |  |  | .092 |
| No | 23532 (98.4) | 1058 (97.1) | 22474 (98.5) |  | 21616 (96.4) | 709 (97.6) | 20907 (96.4) |  |
| Yes | 372 (1.6) | 34 (2.9) | 338 (1.5) |  | 816 (3.6) | 18 (2.4) | 798 (3.6) |  |
| Laxatives or diuretics |  |  |  | .014 |  |  |  | .413 |
| No | 23602 (98.7) | 1065 (97.7) | 22537 (98.7) |  | 21981 (98) | 711 (97.5) | 21270 (98.1) |  |
| Yes | 302 (1.3) | 27 (2.3) | 275 (1.3) |  | 451 (2) | 16 (2.5) | 435 (1.9) |  |
| Vomiting after eating |  |  |  | .002 |  |  |  | .039 |
| No | 23441 (98) | 1052 (96.6) | 22389 (98.1) |  | 21701 (96.8) | 694 (95.4) | 21007 (96.9) |  |
| Yes | 463 (2) | 40 (3.4) | 423 (1.9) |  | 731 (3.2) | 33 (4.6) | 698 (3.1) |  |
| Monotrophic diet |  |  |  | <.001 |  |  |  | .598 |
| No | 22579 (94.5) | 987 (91) | 21592 (94.7) |  | 20050 (89.5) | 637 (88.9) | 19413 (89.5) |  |
| Yes | 1325 (5.5) | 105 (9) | 1220 (5.3) |  | 2382 (10.5) | 90 (11.1) | 2292 (10.5) |  |
| Herbal medicines |  |  |  | .007 |  |  |  | .961 |
| No | 22820 (95.4) | 1018 (93.5) | 21802 (95.5) |  | 21377 (95.2) | 692 (95.1) | 20685 (95.2) |  |
| Yes | 1084 (4.6) | 74 (6.5) | 1010 (4.5) |  | 1055 (4.8) | 35 (4.9) | 1020 (4.8) |  |
| Dietary supplements |  |  |  | .475 |  |  |  | <.001 |
| No | 21792 (90.9) | 1002 (91.6) | 20790 (90.9) |  | 18642 (83) | 643 (88.7) | 17999 (82.8) |  |
| Yes | 2112 (9.1) | 90 (8.4) | 2022 (9.1) |  | 3790 (17) | 84 (11.3) | 3706 (17.2) |  |

Values are presented as unweighted frequency (weighted column proportion) and p values were obtained using chi-squared tests with Rao-Scott correction. Sample weights were integrated and used, in all analyses performed for male and female students, respectively.

**Supplement 2. Comparison of weight control methods according to body mass index**

| **Variables for weight control method** | **Male students** | | | | **Female students** | | | |
| --- | --- | --- | --- | --- | --- | --- | --- | --- |
|  | **Body mass index** | | |  | **Body mass index** | | |  |
|  | **Total** | **Overweight** | **Obese** | ***P* value** | **Total** | **Overweight** | **Obese** | ***P* value** |
| Regular exercise |  |  |  | <.001 |  |  |  | .494 |
| No | 4959 (20.9) | 2167 (19.6) | 2792 (21.9) |  | 6222 (28) | 3512 (28.2) | 2710 (27.7) |  |
| Yes | 18945 (79.1) | 8811 (80.4) | 10134 (78.1) |  | 16210 (72) | 9203 (71.8) | 7007 (72.3) |  |
| Fasting for at least 24 hours |  |  |  | <.001 |  |  |  | <.001 |
| No | 21914 (91.6) | 10149 (92.4) | 11765 (91) |  | 19857 (88.8) | 11368 (89.6) | 8489 (87.7) |  |
| Yes | 1990 (8.4) | 829 (7.6) | 1161 (9) |  | 2575 (11.2) | 1347 (10.4) | 1228 (12.3) |  |
| Eating less |  |  |  | <.001 |  |  |  | <.001 |
| No | 4759 (19.6) | 2552 (22.8) | 2207 (16.9) |  | 1775 (7.8) | 1106 (8.5) | 669 (6.8) |  |
| Yes | 19145 (80.4) | 8426 (77.2) | 10719 (83.1) |  | 20657 (92.2) | 11609 (91.5) | 9048 (93.2) |  |
| Prescription weight loss medication |  |  |  | <.001 |  |  |  | <.001 |
| No | 23451 (98) | 10823 (98.5) | 12628 (97.7) |  | 21751 (96.9) | 12439 (97.8) | 9312 (95.7) |  |
| Yes | 453 (2) | 155 (1.5) | 298 (2.3) |  | 681 (3.1) | 276 (2.2) | 405 (4.3) |  |
| Over-the-counter weight loss medication |  |  |  | .175 |  |  |  | <.001 |
| No | 23532 (98.4) | 10831 (98.5) | 12701 (98.3) |  | 21616 (96.4) | 12341 (97.1) | 9275 (95.5) |  |
| Yes | 372 (1.6) | 147 (1.5) | 225 (1.7) |  | 816 (3.6) | 374 (2.9) | 442 (4.5) |  |
| Laxatives or diuretics |  |  |  | .735 |  |  |  | .828 |
| No | 23602 (98.7) | 10844 (98.7) | 12758 (98.6) |  | 21981 (98) | 12453 (98.1) | 9528 (98) |  |
| Yes | 302 (1.3) | 134 (1.3) | 168 (1.4) |  | 451 (2) | 262 (1.9) | 189 (2) |  |
| Vomiting after eating |  |  |  | .841 |  |  |  | .651 |
| No | 23441 (98) | 10764 (98.1) | 12677 (98) |  | 21701 (96.8) | 12301 (96.8) | 9400 (96.9) |  |
| Yes | 463 (2) | 214 (1.9) | 249 (2) |  | 731 (3.2) | 414 (3.2) | 317 (3.1) |  |
| Monotrophic diet |  |  |  | .019 |  |  |  | .264 |
| No | 22579 (94.5) | 10420 (95) | 12159 (94.2) |  | 20050 (89.5) | 11391 (89.7) | 8659 (89.2) |  |
| Yes | 1325 (5.5) | 558 (5) | 767 (5.8) |  | 2382 (10.5) | 1324 (10.3) | 1058 (10.8) |  |
| Herbal medicines |  |  |  | .452 |  |  |  | <.001 |
| No | 22820 (95.4) | 10505 (95.5) | 12315 (95.3) |  | 21377 (95.2) | 12210 (95.9) | 9167 (94.2) |  |
| Yes | 1084 (4.6) | 473 (4.5) | 611 (4.7) |  | 1055 (4.8) | 505 (4.1) | 550 (5.8) |  |
| Dietary supplements |  |  |  | <.001 |  |  |  | <.001 |
| No | 21792 (90.9) | 10114 (91.7) | 11678 (90.3) |  | 18642 (83) | 10744 (84.4) | 7898 (81.1) |  |
| Yes | 2112 (9.1) | 864 (8.3) | 1248 (9.7) |  | 3790 (17) | 1971 (15.6) | 1819 (18.9) |  |

Values are presented as unweighted frequency (weighted column proportion) and p values were obtained using chi-squared tests with Rao-Scott correction. Sample weights were integrated and used, in all analyses performed for male and female students, respectively.
